# Coping profiles and subjective well-being among people living with HIV: less intensive coping corresponds with better well-being

**DOI:** 10.1007/s11136-017-1612-7

**Published:** 2017-06-05

**Authors:** Marcin Rzeszutek, Ewa Gruszczyńska, Ewa Firląg-Burkacka

**Affiliations:** 1 Faculty of Psychology, University of Warsaw, Stawki 5/7, 00-183 Warsaw, Poland; 2 Health Psychology Department, University of Social Sciences and Humanities, Chodakowska 19/31, 03-815 Warsaw, Poland; 3 Warsaw’s Hospital of Infectious Diseases, Wolska 37, 01-201 Warsaw, Poland

**Keywords:** HIV, Subjective well-being, Stress coping, Latent profile analysis

## Abstract

**Purpose:**

The aim of this study was to investigate the relationship between coping strategies and subjective well-being (SWB) among people living with HIV (PLWH) using the latent profile analysis (LPA) with control for socio-medical covariates.

**Methods:**

The sample comprised five hundred and thirty people (*N* = 530) with a confirmed diagnosis of HIV+. The study was cross-sectional with SWB operationalized by satisfaction with life (Satisfaction with Life Scale) and positive and negative affect (PANAS-X). Coping with stress was measured by the Brief COPE Inventory, enriched by several items that assessed rumination and enhancement of positive emotional states. Additionally, the relevant socio-medical variables were collected.

**Results:**

The one-step model of LPA revealed the following: (1) a solution with five different coping profiles suited the data best; (2) socio-medical covariates, except for education, were not related to the profiles’ membership. Further analysis with SWB as a distal outcome showed that higher intensity coping profiles have significantly worse SWB when compared with lower intensity coping profiles. However, the lowest SWB was noted for mixed intensity coping profile (high adaptive/low maladaptive).

**Conclusions:**

The person-centered approach adopted in this study informs about the heterogeneity of disease-related coping among PLWH and its possible reactive character, as the highest SWB was observed among participants with the lowest intensity of coping.

The literature on coping among people living with HIV (PLWH) is large but highly heterogeneous with regard to coping measurements, coping outcomes, and final remarks [1]. The vast majority of studies on this topic have concentrated on the role of active and avoidant coping. While active coping is related to greater level of CD4-cell counts [2], fewer HIV-related symptoms [3], better quality of life [4], lower frequency of alcohol and drug use [5] and better adherence to treatment [6], avoidant coping have been associated with deterioration of psychosocial and health status of PLWH, including worse physical functioning [7], poor quality of life [8], frequent use of alcohol and drugs [5], and non-adherence to treatment [6]. More specifically, Moskowitz et al. [9] found that meaning-focused coping was consistently linked with better affective, behavior, and physical health outcomes among PLWH. McIntosh and Rosselli [10] observed that spiritual coping and positive reframing promoted psychological adaptation among HIV-infected women to a greater degree than social support seeking. Furthermore, Kraaij et al. [11] noted that cognitive coping strategies (e.g., positive reappraisal) and a proper goal adjustment (e.g., disengagement from unrealistic goals, and reengaging in alternative meaningful aims) have the strongest impact on well-being among HIV-infected men.

However, even if there is a generally accepted consensus that coping matters with regard to how people deal with adverse life events, little agreement has been reached on how to conceptualize, measure, and classify different ways of coping [12–14]. One of the central problems in coping literature is that many authors neglect that the term “coping” is not a unique, observable behavior, stable trait, or easily reported specific belief. On the contrary, it is a dynamic, multidimensional construct that encompasses various actions, behaviors, emotions, and cognitions often used by the same person simultaneously [15, 16]. Finally, the vast majority of authors define ways of coping according to a variable-oriented approach, in terms of dimensions, whereas the fact that the same person may have different positions on each dimension is disregarded [17, 18].

In that light, the central question is not whether some ways of coping are less or more effective, but rather how a specific person copes with a particular stressor and what his or her effectiveness is in doing so. Therefore, a person-centered approach is likely to bring a new perspective to examine coping complexities [19]. It is particularly important since some studies have already demonstrated that a higher intensity of coping may be related to worse, instead of better adaptation (e.g., [20, 21]) and the idea of the possibly defensive nature of intense coping was introduced by Krohne [22] more than two decades ago. Nevertheless, it is worth mentioning that this conceptualization aligns with the definition of coping as individuals’ efforts to reduce imbalance between demands and resources, provided by Folkman and Lazarus [23]. When those efforts are highly diversified and all of them are performed with a high intensity, regardless of specific situational demands, they may actually be a sign of high distress or, in other terms, an indicator of a strong conflict between demands and available resources. When such intensity of coping is performed without goodness of fit to the situational demands for a longer time, it may lead to significant psychological consequences. First, it shows that the aforementioned imbalance has not been resolved despite the efforts made, so the person is still under stress. Second, keeping up such efforts is not possible without costs, which may additionally influence subjective well-being (SWB).

Numerous studies have been conducted on the concept of well-being not only in psychology, but also in other social sciences (e.g., [24–26]). Nevertheless, the question of how well-being should be defined and operationalized still remains largely unresolved [27, 28]. Despite various approaches to well-being, a majority of the authors agree that well-being is a multidimensional construct and there is a necessity to be clear about what is being measured [29, 30]. In this study, we concentrated on SWB defined broadly by the level of satisfaction with life and a combination of positive and negative affect [31–33] among people living with HIV (PLWH). The issue of SWB seems to be of special interest among patients dealing with chronic disease, with significant psychological and social burden, such as PLHW. The substantial progress in antiretroviral therapy has changed social attitudes toward HIV/AIDS from a fatal and terminal illness to a chronic medical condition and has given great hope to PLWH for a longer life [34, 35]. However, PLWH still experience major psychological distress stemming from being diagnosed with a potentially life-threatening virus [36, 37], unpredictability of HIV symptoms fluctuation [38, 39], and social isolation and discrimination [40, 41]. Not only can HIV-related distress deteriorate SWB, but poor SWB among this patient group impacts the course of HIV infection by diminishing CD4 cell counts, which influence the pace of HIV progression [42]. Therefore, research on SWB among PLWH has important clinical implications [43, 44]. Nevertheless, the majority of studies on SWB among PLWH concentrated solely on the presence or absence of these negative HIV-related mental health problems (e.g., [45, 46]). Therefore, in this study, we focused on the aforementioned broad definition of SWB among PLWH.

## Current study

Despite the same medical diagnosis and controlling for other medical variables, there is a significant interindividual variability in coping with HIV infection [1]. Further, although the results from the variable-centered studies seem reasonably coherent, they do not take into account these individual differences, that is, each person may perform a different combination of so-called adaptive and maladaptive strategies. To address this gap, we implemented a person-centered approach using a latent profile analysis (LPA). Thus, the aim of the study was twofold: First, to investigate heterogeneity of coping with the disease among PLWH, including possible socio-medical covariates; and second, to examine whether different coping profiles are related to SWB in this patient group [47].

On the basis of the aforementioned studies on coping and SWB among PLWH, we generated three specific hypotheses. First, we expected that the sample was heterogeneous in terms of coping, i.e., different coping profiles can be observed among the participants. Second, allocation to a specific coping profile is related to socio-medical status since this status may serve as a proxy of current level of resources available to the person [48]. Finally, we assumed that higher intensity coping profiles are related to worse SWB when compared with lower intensity coping profiles. Additionally, participants belonging to the mixed intensity coping profile (higher intensity on some coping strategies and lower on others) have higher SWB. More specifically, for mixed profiles, a combination of higher intensity of strategies related to more adaptive outcomes (e.g., active and problem-focused coping, including positive reframing and positive emotional enhancement) and lower intensity of so-called maladaptive strategies (e.g., avoidant coping and palliative forms of emotion-focused coping) should be the best in terms of well-being [49].

## Method

### Participants and procedure

Five hundred and thirty (530) adults with a medical diagnosis of HIV infection were recruited from patients of the out-patient clinic of the state hospital for infectious diseases. The participants completed a paper-and-pencil version of the measures and participated in the study voluntary (there was no remuneration). The eligibility criteria were that participants had to be 18 years of age or older, had to have a medically confirmed diagnosis of HIV+, and had to be a recipient of antiretroviral treatment at the out-patient clinic where the study was conducted. The exclusion criteria included HIV-related cognitive impairment diagnosed by medical doctors. In particular, out of the 750 patients eligible for the study, 530 were approached and agreed to the filed measures (71%), 152 declined (20%), and 68 (9%) had missing data to an extent that precluded them from the analysis [50].

Specifically, there were 444 men (84%) and 86 women (16%) between 18 and 76 years of age (*M* = 39.81; SD = 10.54) of whom 57% of were married. Only 16% of the participants had elementary education, 31% reported secondary and 53% higher education. Majority of the participants, declared full employment (72%), 12% were unemployed, 12% were receiving a pension, and 4% were retired. When it came to the clinical variables, the HIV infection duration ranged from 1 to 32 years (*M* = 7.71; SD = 6.86). The antiretroviral treatment duration ranged from 1 to 32 years (*M* = 5.97; SD = 5.53), and the CD4 cell count ranged from 100 to 2,000 (*M* = 589.46; SD = 222.42). Finally, out of the whole sample, 15% patients were diagnosed with AIDS.

## Measures

### Subjective well-being indicators

SWB was measured on the Satisfaction with Life Scale (SWLS; [31] along with the Positive and Negative Affect (PANAS-X; Watson and Clark [51]. The SWLS comprises five items, each with a seven-point scale, ranging from 1 (*strongly disagree*) to 7 (*strongly agree*). A higher total score means a higher level of satisfaction with life. The Cronbach’s alpha in the current study was satisfactory (.88). The PANAS-X comprises 10 adjectives for positive affect (e.g., *proud*, *excited*, etc.) and 10 for negative affect (e.g., *frightened*, *hostile*, etc.). The participants were asked to rate their general affective states on a five-point response scale from 1 (*not at all*) to 5 (*extremely*). The Cronbach’s alpha coefficients obtained in this study were .85 for the positive affect subscale and .86. for the negative affect subscale.

### Coping strategies

To assess strategies for coping with stress, the Brief COPE Inventory was used [52]. This tool consists of 28 items and provides 14 subscales with a different reliability, two items each with a Likert-like response scale ranging from 0 (*I haven’t been doing this at all*) to 3 (*I’ve been doing this a lot*). Coping intensity is understood as participants’ self-report on the magnitude with which their use a given strategy to deal with health issues caused by being infected with HIV. The subscales were derived empirically, and they were not theoretically reassessed afterwards to propose a more comprehensive systematization of coping strategies (see [14]). In particular, as this tool does not include items directly referring to rumination, which is one of the most strongly proved maladaptive strategies (see [53]), and items describing coping efforts focused on enhancement of positive emotional states during stress, the relevant two items were added from the Ruminative Response Styles [54]: *I think What am I doing to deserve this?*; *I think Why do I have problems other people don’t have?*) and from the Coping with Health Injuries and Problems Scale after modification [55]: *I have nice things around*; *I look for simple pleasures (e.g., having a cup of tea, listening to music, walking, reading a good book)*). Therefore, there were 16 coping indicators in the study: self-distraction, active coping, denial, substance use, use of emotional support, use of instrumental support, behavioral disengagement, venting, positive reframing, planning, humor, acceptance, religion, self-blame, rumination, and positive emotion enhancement. The Cronbach’s alpha ranged from .78 to .86. Due to reasons described in the introduction, we did not classify the subscales into the higher order coping indices, as this kind of aggregation may influence segmentation results.

### Data analysis

LPA is a statistical method that enables investigation of unobserved heterogeneity within a studied sample, that is, it identifies groups of participants who represent the greatest similarity on the same set of observed continuous variables within a given group and the greatest dissimilarity between other participants’ groups [56]. In our study, this method allows classifying participants into a number of exclusive and exhaustive subgroups, characterized by different coping profiles. A model with an optimal number of such categories (i.e., profiles) is selected on the basis on several indicators. For Akaike’s information criterion (AIC), Bayesian information criterion (BIC), and the sample-size adjusted BIC (SABIC), lower values indicate a model with better fit [57]. Another evaluation of goodness of fit is provided by the results of the bootstrap likelihood ratio test (BLRT [58], which compares neighboring models. An entropy-based criterion indicates a quality of a class separation from 0 to 1, where 1 evidences a perfect classification [19]. Finally, a size of the smallest class is a practical criterion, since classes smaller than 5% of the sample is considered spurious and unreplicable [59].

In general, we used LPA with distal outcomes [60]. First, to select a model with an optimal number of coping profiles, we adopted the one-step approach with socio-medical covariates (gender, age, marital status, education, employment, HIV/AIDS status, HIV infection duration in years, antiretroviral treatment duration in years, CD4 count) included in the process of segmentation, thus the obtained coping profiles were adjusted in this regard [61]. Then we regressed a distal outcome, that is, SWB, on latent coping profiles using the bias-adjusted three-step analysis described by Vermunt and Magidson [62, 63]. The calculations were performed using the Latent GOLD 5.1 (containing a submodule called Step3) and IBM SPSS Statistics version 24.

## Results

### Descriptive statistics

Mean values, standard deviations, and Pearson‘s correlations of the main study variables are presented in Table 1.

**Table 1:** Descriptive statistics and Pearson’s correlations of the study variables (*N* = 530)

Variable		M	SD	Range	Kurtosis	Skewness	1	2	3	4	5	6	7	8	9
1	Positive affect	3.44	0.65	1–5	−0.03	−0.33	1								
2	Negative affect	2.40	1.01	1–5	−0.84	0.46	−.02	1							
3	Satisfaction with Life	20.31	6.39	5–35	−0.39	−0.38	.42*	−.34*	1						
4	Active coping	3.46	1.26	0–6	0.41	−0.47	.02	.04	.12*	1					
5	Planning	3.60	1.34	0–6	0.43	−0.51	.03	−.03	.03	.48*	1				
6	Positive reframing	3.55	1.30	0–6	0.58	−0.59	.05	.00	.06	.26*	.42*	1			
7	Acceptance	3.93	1.19	0–6	0.76	−0.41	.07	−.15*	.13*	.20*	.42*	.33*	1		
8	Humor	2.83	1.41	0–6	−0.41	0.06	.10*	.13*	−.02	.14*	.28*	.39*	.20*	1	
9	Religion	2.40	1.87	0–6	−1.13	0.14	−.02	.19*	−.02	.21*	.20*	.27*	.14*	.49*	1
10	Use of emotional support	2.69	1.57	0–6	−0.68	0.05	.07	.21*	−.01	.36*	.37*	.34*	.10*	.43*	.37*
11	Use of instrumental support	3.40	1.35	0–6	0.12	−0.42	.10*	.12*	.04	.39*	.42*	.39*	.26*	.39*	.38*
12	Self-distraction	3.02	1.29	0–6	−0.08	−0.35	.01	.21*	−.03	.39*	.23*	.21*	.15*	.29*	.29*
13	Denial	2.22	1.62	0–6	−0.71	0.25	−.11*	.34*	−.17*	.26*	.15*	.20*	−.06	.37*	.42*
14	Venting	2.93	1.42	0–6	−0.31	−0.35	−.11*	.26*	−.15*	.26*	.30*	.24*	.18*	.42*	.37*
15	Substance use	2.18	1.83	0–6	−1.09	0.26	−.06	.35*	−.16*	.17*	.11*	.18*	−.06	.39*	.41*
16	Behavioral disengagement	2.33	1.61	0–6	−0.66	0.24	−.14*	.36*	−.19*	.20*	.10*	.21*	−.10	.44*	.47*
17	Self-blame	2.85	1.57	0–6	−0.72	−0.23	−.21*	.34*	−.22*	.20*	.27*	.16*	.03	.31*	.43*
18	Rumination	2.76	1.75	0–6	−0.76	0.00	−.14*	.29*	−.16*	.21*	.25*	.17*	.11*	.45	.49*
19	Positive emotion enhancement	3.90	1.36	0–6	0.72	−0.58	.09	−.03	.13*	.23*	.39*	.39*	.40*	.29*	.20*

Variable		M	SD	Range	Kurtosis	Skewness	10	11	12	13	14	15	16	17	18
1	Positive affect	3.44	0.65	1–5	−0.03	−0.33									
2	Negative affect	2.40	1.01	1–5	−0.84	0.46									
3	Satisfaction with Life	20.31	6.39	5–35	−0.39	−0.38									
4	Active coping	3.46	1.26	0–6	0.41	−0.47									
5	Planning	3.60	1.34	0–6	0.43	−0.51									
6	Positive reframing	3.55	1.30	0–6	0.58	−0.59									
7	Acceptance	3.93	1.19	0–6	0.76	−0.41									
8	Humor	2.83	1.41	0–6	−0.41	0.06									
9	Religion	2.40	1.87	0–6	−1.13	0.14									
10	Use of emotional support	2.69	1.57	0–6	−0.68	0.05	1								
11	Use of instrumental support	3.40	1.35	0–6	0.12	−0.42	.56*	1							
12	Self-distraction	3.02	1.29	0–6	−0.08	−0.35	.30*	.33*	1						
13	Denial	2.22	1.62	0–6	−0.71	0.25	.52*	.27*	.39*	1					
14	Venting	2.93	1.42	0–6	−0.31	−0.35	.49*	.40*	.40*	.56*	1				
15	Substance use	2.18	1.83	0–6	−1.09	0.26	.46*	.28*	.28*	.61*	.50*	1			
16	Behavioral disengagement	2.33	1.61	0–6	−0.66	0.24	.54*	.28*	.38*	.70*	.53*	.61*	1		
17	Self-blame	2.85	1.57	0–6	−0.72	−0.23	.36*	.21*	.33*	.55*	.51*	.48*	.56*	1	
18	Rumination	2.76	1.75	0–6	−0.76	0.00	.40*	.34*	.30*	.52*	.49*	.41*	.50*	.59^*^	1
19	Positive emotion enhancement	3.90	1.36	0–6	0.72	−0.58	.14*	.31*	.20*	.03	.18*	−.01	.02	.10*	.29*

All the correlations marked with asterisk are significant at least at *p* < .05; *M* mean, *SD* standard deviation

All the variables can be regarded as normally distributed. Positive affect and negative affect were uncorrelated (*r* = −.03) and only up to medium were they related to satisfaction with life. Thus, it indicates that they these domains of SWB are indeed separate to a significant degree. Among coping strategies, the highest correlation was noted for denial and behavioral disengagement (*r* = .70).

### Coping profiles and their socio-medical correlates

Table 2 summarizes the indices of the model selection process for one to six profile solutions. BIC, AIC, and SABIC (see, "Data analysis") indicate on six-profile model. Also, BLRT informs that adding a profile to each consecutive model significantly improves goodness of fit which may also point at the most numerous profile solutions. Entropy values were similar for all the models so each of them provides a good separation in the sample.

**Table 2:** Summary of model selection indices of latent prolife analysis

Model	BIC	AIC	SABIC	Number of parameters	Entropy	BLRT		Smallest profile	
						value	*p*	% of *N*	frequency
1-Profile	30788.37	30651.64	30686.79	32					
2-Profile	29244.56	29001.01	29063.63	57	0.89	1700.63	<.001	42	221
3-Profile	28816.77	28466.37	28556.46	82	0.87	584.63	<.001	17	92
4-Profile	28487.93	28030.74	28148.288	107	0.87	485.64	<.001	12	64
5-Profile	28383.92	27819.90	27964.91	132	0.86	260.84	<.001	9	47
6-Profile	28291.77	27620.92	27793.40	157	0.87	248.95	<.001	3	17

*BIC* Bayesian information criterion, *AIC* Akaike’s information criterion, *SABIC* sample-size adjusted BIC, *BLRT* bootstrap likelihood ratio test

**Fig. 1:**
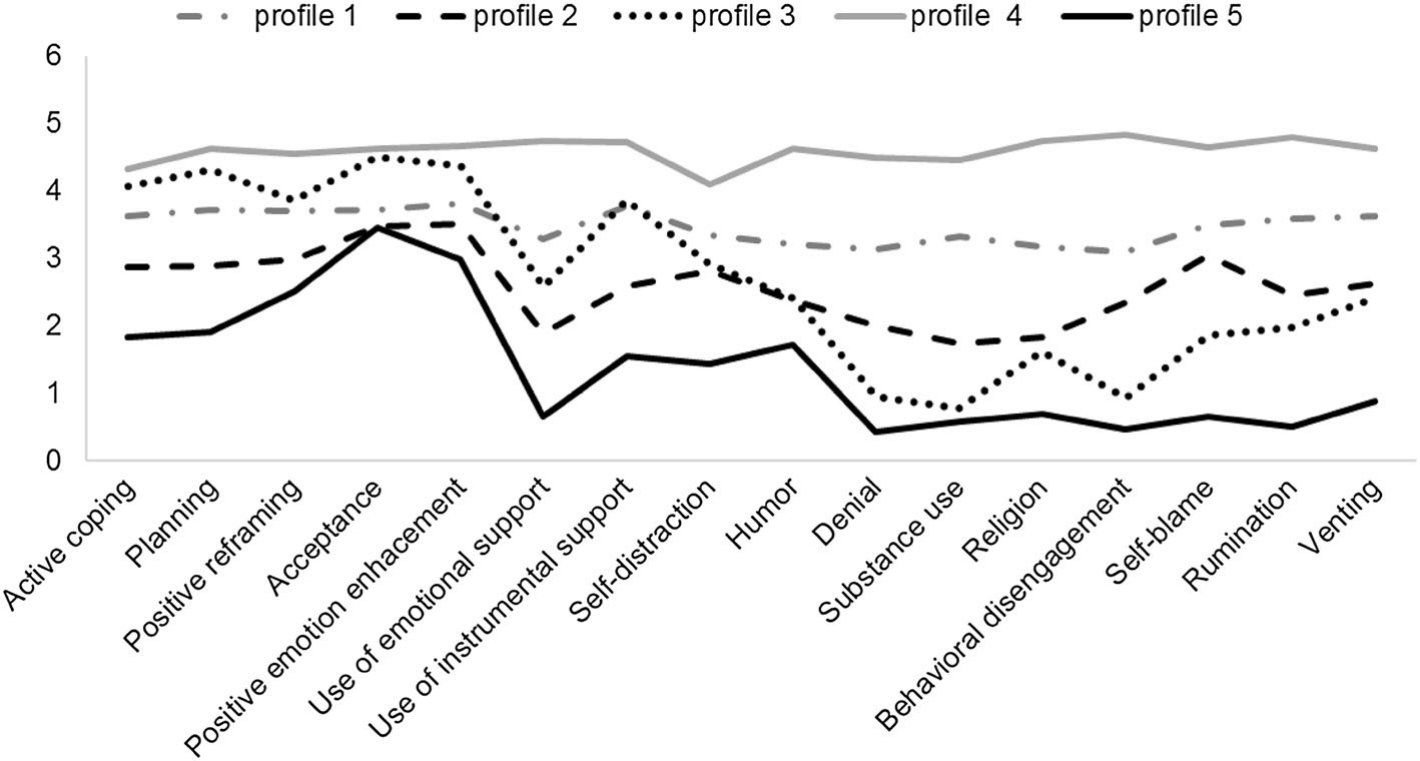
Results of latent profile analysis: five coping profiles identified in the studied sample (*N* = 530)

However, the size of the smallest group in the six-profile solution was as low as 3% of the sample. Thus, the second best fitted model, a five-profile solution, was chosen for further analysis. Figure 1 illustrates this model.

As it can be seen, four out of five coping profiles are mostly parallel, whereas the remaining one crosses over from high values to lower ones (profile 3). The most numerous group consists of 159 participants (30%, profile 1) and can be described as high intensity coping profile. The second one, represented by 135 participants (25.5%, profile 2), has a generally lower profile, especially with regards to denial, substance use and turn to religion strategies. As already mentioned, mixed profile was observed for 130 participants (24.5%, profile 3). They have high values on coping strategies frequently named as adaptive coping and lower values on strategies named maladaptive coping. The highest intensity coping profile is represented by 59 participants (11%, profile 4). Finally, the smallest group with the lowest intensity coping profile included 47 participants (9%, profile 5). It is worth noticing, however, that the highest and the lowest profiles have a similar number of members. Also, profiles for intense copers (profile 1 and profile 4) are more flatted whereas profiles for mild copers (profile 2 and 5) have slightly higher values in positive reframing, acceptance and enhancement of positive emotions. The averaged posterior probability ranged from .89 for the mixed profile to.97 for the most intensive coping profile.

Finally, it turned out that profiles differed only in terms of education (Wald = 11.31, *p* = .02). The pairwise comparisons revealed that the highest proportion of patients with university degree was in profile 2 (2.28) and 5 (1.35), while in other profiles this distribution was statistically equal for all the levels of education. Therefore, the coping profiles’ membership appeared to be relatively independent of socio-medical variables, which was also reflected in standard R squared for a covariate-based classification equaled only .03.

### Relations of coping profiles with well-being

Significant differences between coping profiles with regard to SWB were noted. Table 3 presents test values and means for each profile for illustrative purposes.

**Table 3:** Results of the bias-adjusted step-three analysis for coping profiles and subjective well-being as a distal outcome

Distal outcome	Wald	*p*	Mean				
			Profile 1	Profile 2	Profile 3	Profile 4	Profile 5
Satisfaction with life	72.85	<.001	19.18	23.13	17.69	19.98	23.82
Positive affect (PA)	50.17	<.001	3.39	3.66	3.15	3.45	3.76
Negative affect (NA)	140.05	<.001	2.93	1.84	2.48	2.66	1.68
PA/NA			1.34	2.23	1.45	1.68	2.59

Additionally, to elaborate on affective well-being, a positive affect to negative affect ratio was added. As can be seen, the highest satisfaction with life and positive affect as well as the lowest negative affect was noted for profile 5 and then for profile 2, thus the *lowest* profiles have the *highest* SWB. Interestingly, the lowest SWB was observed among participants with a mixed intensity coping profile.

## Discussion

The results of our study were consistent with the first hypothesis, that is, we observed the heterogeneity of the sample of PLWH with regard to coping with the disease, as five different coping profiles were observed. Thus, our findings are in line with ideas expressed a long time ago, as well as with contemporary critical observations on the nature of coping that it is a multidimensional construct that operates on the number of different levels [64, 22, 65]. As far as PLWH is concerned, our results may be interesting in and of itself, as till now the vast majority of studies among PLWH were conducted in the variable-oriented model, and were focused on searching for single sociodemographic or medical variables that are independently associated with various coping strategies, neglecting the problem of heterogeneity of coping in this group of patients [45, 43, 1]. The domination of variable-oriented model may also be the reason why ambiguous results on well-being among PLWH exist in the literature. According to Keiser et al. [66], many authors disregard how particular socio-medical and psychosocial variables cluster across different PLWH subgroups distinguished on the basis of various SWB profiles, which can be influenced by a large number of factors simultaneously. Our study addressed these two gaps in the literature by examining both coping profiles and subjective well-being among people living with HIV using latent profile analysis.

Secondly, socio-medical variables, with the exception of education, were not related to the coping profiles among our participants and, as such, were also irrelevant for observed SWB differences between these profiles, which contradicted our second hypothesis. This finding is contrary to several previous studies, which showed that coping among HIV+ individuals is shaped greatly by clinical variables, such as a CD4 cell count [67, 68], HIV infection duration [37], being diagnosed with AIDS in particular [69], being on ART treatment [70, 71] or sociodemographic data [46]. In our sample, however, coping profiles and consequently SWB differences as well were related only to having a university degree. This finding may be discussed in the context of existing HIV-related stigma, and threat of social rejection, including in particular losing social status, which is still a very prevalent experience among PLWH [40, 72]. Perhaps higher education acts here as a personal resource that offers an opportunity for a greater social participation and reduces stress level both directly through a lower number of stressors and indirectly through more effective but less intense coping [73]. Therefore, education may be a better proxy of health-related stress and well-being than clinical variables since the great advancements in HIV/AIDS treatment have improved substantially the life expectancy of PLWH [35]. As a result, an increasing number of PLWH are more concerned with their social status not only with health outcomes. It is also in accordance with other studies, which indicated that subjective well-being among chronically ill patients depends more on psychosocial factors rather than socio-medical variables [74].

Finally, in line with our third hypothesis, higher intensity coping profiles were related to worse well-being when compared with lower intensity coping profiles, but contrary to our expectations, members of the mixed intensity profile (high adaptive/low maladaptive) have the lowest SWB. This result is particularly intriguing, as there is a common assumption in coping literature that more intense adaptive coping provides better effects for individual’s well-being across different stressful situations (e.g., [75–77]. Again, perhaps there is a need to come back to basics, as Krohne [22] previously suggested that low- intensity coping and diversity may just mean a low level of distress. There is the theoretical argument that coping is a psychological necessity only when a person is under stress according to his or her cognitive appraisal. However, at least for some, their extensive coping efforts do not bring any relief in this regard. In uncontrollable situations, such as terminal disease, coping may even be purely reactive, that is, the process of coping may be initiated only due to experiencing strong negative emotions, no matter if this specific way of coping is meaningful in this situation or not [78]. One study regarding PLWH provided data in accordance with our findings. Fleishman et al. [79] in a study on coping in response to HIV/AIDS proved that PLWH classified as passive copers had fewer HIV-related symptoms, a better level of physical functioning, and high affective well-being. It is likely that in an uncontrollable situation, and many aspects of being HIV+ can be described as such, intensive coping may elevate more HIV-related distress, but again, there is no consensus on that in the literature [1].

However, our study is not free of limitations. First of all, the cross-sectional design prevents us from making causal interpretations and future studies should be conducted in a longitudinal design. Even if well-being was an explanatory variable here, the possibility that its low values may be a source of stress itself, thus a cause and not an outcome of coping, cannot be excluded. Secondly, we did not control for the level of stress experienced by the participants. In future research, the intensity of stress should be treated as a covariate, e.g., participants that experienced more level of stress may have higher coping intensity. Thirdly, we assessed, a broad but still selective set of coping strategies, so it would be advisable in the future to check if the obtained effects are present for other coping measurements. Finally, in our sample significant underrepresentation of women is seen, but the gender ratio was typical for PLWH population [80] and this is in line with other studies pointing at a lack of gender differences in coping among PLWH [81, 82].

From a clinical point of view, our findings suggest that when providing psychological help special focused should be put on patients with very intense coping. Furthermore, a modification of coping into so-called favorable profile (high intensity of adaptive strategies accompanied by low intensity of maladaptive strategies) may not necessary is a proper direction. Thus, further research is needed as knowledge about psychological functioning of PLWH is still limited.

## Conclusion

The person-centered approach adopted in this study informs about the heterogeneity of disease-related coping among PLWH and its possible reactive character, as the highest SWB, was observed among participants with the lowest intensity of coping. The results of this study illustrate how a person-centered approach may influence clinically relevant knowledge regarding the complexities of dealing with chronic disease as well as elucidate coping research in general. We have to remember that beyond the coping strategies, there is always the person who copes.
